# Deficits in Working Memory and Theory of Mind May Underlie Difficulties in Social Perception of Children with ADHD

**DOI:** 10.1155/2021/3793750

**Published:** 2021-08-28

**Authors:** Samane Imanipour, Mahmood Sheikh, Monir Shayestefar, Tourandokht Baloochnejad

**Affiliations:** ^1^Department of Sport Science, Faculty of Literature and Humanities, Jahrom University, Jahrom, Iran; ^2^Faculty of Physical Education and Sport Sciences, University of Tehran, Tehran, Iran; ^3^Department of Neuroscience, Faculty of Advanced Technology in Medicine, Iran University of Medical Sciences, Tehran, Iran; ^4^Sport Medicine Research Center, Neuroscience Institute, Tehran University of Medical Sciences, Tehran, Iran; ^5^Department of Physiology, Faculty of Medicine, Iran University of Medical Sciences, Tehran, Iran

## Abstract

Children with attention deficit hyperactivity disorder (ADHD) are prone to peer rejection and disliking due to difficulties in social perception and interaction. To address social perception impairments in ADHD, we examined children with ADHD in a noisy biological motion (BM) direction discrimination paradigm in association with sociocognitive factors including emotion regulation, theory of mind (TOM), and working memory compared to healthy controls. Our results showed that children with ADHD were poorer in discriminating BM direction in noisy environments (*F* (1, 36) = 4.655, *p*=0.038). Moreover, a significant correlation was found between working memory and TOM with BM discrimination in an ADHD group (*r* = 0.442, *p*=0.01, and *r* = 0.403, *p*=0.05, respectively). Our findings could suggest that social perception in noisy scenarios may be affected by memory and social cognitive abilities of children with ADHD.

## 1. Introduction

Attention deficit hyperactivity disorder (ADHD) is characterized by multiple deficits in cognitive, behavioral, and psychological domains including emotion regulation (e.g., impulsivity), working memory, and higher-order social cognitive processing, such as theory of mind (TOM) [1–3].

Although social cognition impairment is not accounted as a diagnostic criterion for ADHD [4], impairment in social relationship, particularly peer relationships, is a prominent and inherent feature in children with ADHD [5, 6]. Studies have represented that children with ADHD are approximately four times more likely to be rejected by their peers compared to typical children, even after periods of social contact as brief as a few hours. A wealth of literature has suggested disruptive and offensive behaviors, lack of prosocial behaviors, and emotion dysregulation as influencing factors that can lead to peers' disliking in children with ADHD [7–9]. To understand why children with ADHD experience difficulties in their social relationships, it is important to investigate underlying sociocognitive mechanisms.

Due to the important role of visual processing of body movements or biological motions (BMs) in a dynamic social world [10], in the current study, we administered a method introduced by Johansson for investigating BM perception of children with ADHD [11]. In other words, the ability of humans to extract accurate perceptual information based solely on a human body movement is defined as a hallmark of social cognition and has an immense value for successful and adaptive social behavior as well as nonverbal communication [12]. Furthermore, due to the fact that real social environments are almost covered by a wide variety of noises, BM perception in the noisy condition has been investigated in social cognitive disorders such as autism and schizophrenia [13, 14]. However, there are few studies investigating BM processing in a noisy condition in children with ADHD. In the only study of its kind, Kroger et al. using event-related potential (ERP) reported a reduced and more diffused activation in occipital-temporal regions when observing BM without any noise in children with ADHD compared to typically developing (TD) children [15]. It is worth mentioning that Kroger showed an intact BM detection at the behavioral level in ADHD. However, it is still unclear whether recognition of BM in children with ADHD remains intact in the presence of different levels of noise in the paradigm.

Moreover, successfully distinguishing BM may rely on bottom-up integration of signals from basic visual motion perception along with top-down social cognition [14]. In this vein, evidences have shown that proper performances in BM tasks require both social motion recognition and perceptual assessment of motion features (e.g., direction) which mostly attributed to top-down and bottom-up brain systems, respectively [14]. Nevertheless, there are rare data showing the association of sociocognitive variables with BM perception in noisy conditions in children with ADHD. So, in the current investigation, we aimed to evaluate whether there is any association between emotion regulation, TOM (emotion perception from eye region), working memory, and BM perception in children with ADHD.

It has long been proposed that individuals with ADHD have deficits in regulating their emotions [16]. There are three models depicting the association between ADHD and emotion regulation as the following: (1) emotion dysregulation is a core feature of ADHD, as central as hyperactivity and inattention to the disorder, (2) emotion dysregulation and ADHD both are belonged to different and distinct entities cosegregating with each other, and (3) emotion dysregulation and ADHD are distinct but do have correlated dimensions. In addition to emotion regulation, evidences have shown that emotion recognition from face or eye region (TOM) is also deficient secondary to impaired executive functioning and attentional limitations in individuals with ADHD [17]. TOM refers to the ability to attribute emotions to oneself and to others mostly from the face region. Emotion perception and TOM are the most important domains of social cognition, and such impairments in recognizing different facial features and associating them with a specific emotion decrease the patient's capacity to adapt to the social settings. Besides, there are evidences showing deficits in working memory (i.e., the ability to monitor and modify entering information) in children with ADHD [18]. Regarding the role of working memory in social perception, there are somehow rich evidences showing that the working memory capacity has a prominent role in adaptive perception, experience, and expression of socioemotional behaviors [19, 20]. Thus, working memory deficits might underlie deficits in perceiving social and emotional stimuli in real social settings containing different types of noises in children with ADHD.

Given together, we hypothesized the possible association of deficits in emotion regulation, TOM, and working memory with BM perception impairments in children with ADHD. To examine BM detection, we administered a motion-noise BM paradigm similar to previous studies [14, 21]. In this paradigm, a human action (walking) that was presented as point light dots was utilized as part of the stimulus. Then, visual motion noise was added as the other part of the stimulus and the participant was supposed to guess the action. Furthermore, by changing the level of noise, we were able to directly examine the effect of the perceptual signal strength on perception of BM.

Finally; we aimed to explore the association between BM performance and the mentioned triad of cognitive factors (i.e., emotion regulation, TOM, and working memory) in order to provide supplemental information about the underlying sociocognitive mechanisms involved in social difficulties in children with ADHD.

## 2. Methods

### 2.1. Participants

Twenty-five children with ADHD and twenty-five age and sex-matched TD children (11 boys and 14 girls; ADHD mean age, 9.93; SD, 1.09; control mean age, 9.76; SD = 1.01) participated in this study. ADHD diagnosis was confirmed utilizing DSM-V [22] which was administered by an expert clinician who was not involved in the research process and was not aware of the objectives of the study. Inclusion criteria for both groups of participants were (1) being between the ages of 7 and 12 years old, (2) having IQ score more than 86, and (3) having no history of psychiatric illness. In order to evaluate IQ, subscales of the Wechsler test of intelligence including verbal comprehension, perceptual reasoning, working memory, and speed processing were used in this study. The parents completed written informed consent, and they assented to assign to the study.

### 2.2. Experimental Procedures

Participants performed all the tasks within the same research laboratory. All task procedures were presented on a 15-inch Glossy Widescreen LCD display with a resolution of 1366 × 768 pixels. BM stimuli were implemented in a dark room while a mild light was emitted from the laptop. Participants were supposed to look at the display and keep their heads unmoving while being seated 65 cm apart from the screen. Before starting the experiment, participants completed two test runs of 10 trials including 10-point light motion at 100 percent coherence and 10 BM tests in light noisy screen, to ensure that they understood the tasks.

Observers judged the overall direction of motion while a central and a stationary red color point was appeared. Observers were encouraged to guess the direction when they were not sure about the direction. They were allowed to indicate the direction of motion using their hand. Feedback was not provided after the child's response. The general experiments were run in a two-block order. Each observer completed 30 trials in the BM task and attended in the cognitive assessments' block. The block order varied between individuals and was counterbalanced within and between participant groups.

The entire psychophysical testing took approximately 1 hour for each TD child and 2 hours for each child with ADHD to be completed. In fact, children with ADHD required more time to be focused and to rest between blocks.

### 2.3. BM Paradigm

**Figure 1:**
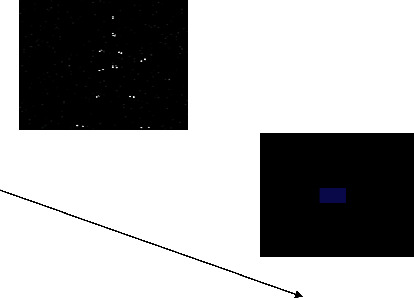
Example of stimulus sequence frames embedded in noise from a biological motion stimulus (bright points inside the pictures) depicting walking. The stimuli were shown for 1 second and then 1 second rest with the blue square.

**Figure 2:**
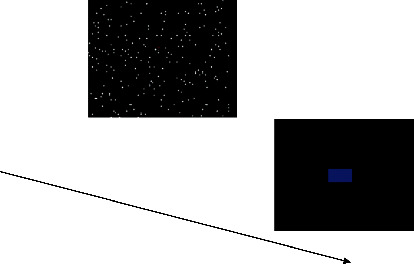
Examples of stimulus sequence. On each trial, subjects were required to judge whether the global motion of the dots was clockwise or counterclockwise of upwards. The stimuli were shown for 1 second and then 1 second rest with the blue square.

Accuracy of BM perception was tested by presenting noise into a standard point-light BM display of a walking human [11]. Each BM target consisted of 13 points of light (black dots on a white background) placed on the major joints and head of a walking figure (each point's size was 5-arc min). This target was embedded in a number of noise dots (duplicated dots from the original biological walkers), and they together constituted a stimulus for perceptual discrimination of BM. The proportion of target points in the BM stimulus is considered the perceptual signal or signal-to-noise ratio. A large percentage of target dots provides a strong perceptual signal and therefore makes the task easier. There were three noise-to-signal ratios of 50%, 75%, and 93% corresponding to moderate, high, and very high noise levels in the stimulus, respectively. The makeup of noise dots was not just in one direction and applied with the following manipulations: half of the motion paths of the noise dots were generated from a walker moving rightward and the other half were generated from a walker moving leftward [14]. This “half and half” makeup modification allowed providing a more balanced noise profile regarding the randomness of motion direction [14] (Figures 1 and 2).

### 2.4. Reading the Mind in the Eyes Test (RMET)

In order to evaluate the participants' ability and to recognize emotional expressions from the eye region, the electronic version of the RMET was administered [23]. This ability is closely associated with TOM, and the RMET has been extensively researched and found to be a reliable measure of TOM [23–25].

The RMET contains 36 images, and each image remains on the screen until a response from the participant is registered. Each image has four options of different emotions, and the participants are supposed to choose the option which best described the emotional expression of the image. Performance was measured by the proportion of accurate responses to the total images.

### 2.5. Working Memory

In order to assess working memory, we administered the Wechsler Intelligence Scale for Children-Forth Edition (WISC-IV). WISC-IV contains four subtests including verbal comprehension index, perceptual reasoning index, working memory index, and processing speed index. The working memory subtests of WISC-IV contain digit span (DS), letter-number sequencing, and arithmetic tests. In the current study, we utilized the DS test in which participants were supposed to hear and then verbally reproduce an increasing number of digit strings (two trials per digit string length) in forward order (DS forward) and backward order (DS backward) [26].

### 2.6. Emotion Regulation

The Emotion Regulation Questionnaire for Children and Adolescents (ERQ-CA) [27] is a 10-item scale designed to measure respondents' tendency to regulate their emotions in two ways of cognitive reappraisal and expressive suppression. Participants were answered each item on a 7-point Likert-type scale ranging from 1 (strongly disagree) to 7 (strongly agree). The ERQ has been reported to have high internal consistency (*a* = 0.79 for reappraisal and 0.73 for suppression) and test-retest reliability (*r* = 0.69 for both scales) (42).

### 2.7. Statistics

We calculated the percentage of accurate responses in discriminating BM direction for each participant. We performed ANOVA in order to compare the accuracy of responses regarding three levels of noise in BM direction discrimination both within and between groups. The *t*-test was conducted to examine the association between BM direction discrimination and scores on emotion dysregulation, RMET, and working memory. The *p* values less than 0.05 were considered as the level of significance. Analyses were performed using SPSS software version 24 (SPSS Inc., Chicago, IL, USA).

## 3. Results

### 3.1. BM Direction Discrimination

**Figure 3:**
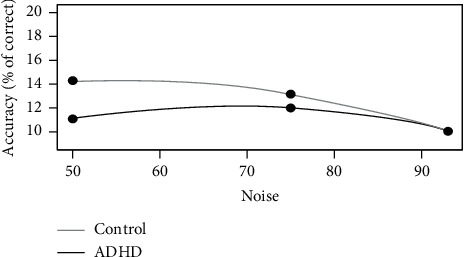
Subjects' performances as a function of noise-to-signal ratio of biological motion. The curve represents the fits of data for three noise situations for normal and ADHD groups.

Our results generally showed that there is no significant difference regarding direction discrimination in BM trials between two groups (the ADHD group vs. the control group: 31.7 vs. 35.2; F (1, 36): 1.42, *p*: 0.24). However, the results of ANOVA indicated significant between-group difference regarding BM direction discrimination in only the moderate noise-to-signal ratios (50% and 75%) (*F* (1, 36) = 4.655, *p*=0.038). Furthermore, within-group analysis showed that among the ADHD participants, there is no significant difference regarding the three noise-to-signal ratios of BM direction discrimination (*F* (1, 18) = 0.048, *p*=0.82). Nevertheless, the results in normal participants represented significant differences regarding BM direction discrimination between the very high and moderate noise-to-signal ratios (*p*=0.001) as well as very high and high ratio (*p*=0.001) (*F* (1, 18) = 14.097, *p*=0.001) (Figure 3).

### 3.2. RMET

We examined the difference in mean scores of the RMET between ADHD and normal groups (10.47 (4.61) and 16.47 (1.89), respectively). The results represented significant difference in the mean of correct responses in the RMET between two groups (*t* (36) = −5.24, *p*=0.01).

### 3.3. Working Memory

We measured working memory (DS task) subtests of WISC-IV in which there was a significant difference between scores of two groups (*t* (36) = −5.082, *p*=0.001).

### 3.4. Emotion Regulation

**Figure 4:**
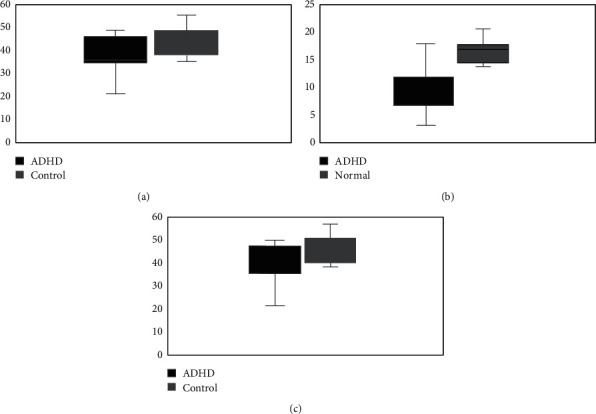
Summary of the performances on (a) emotion regulation, (b) Reading the Mind in the Eyes test, and (c) working memory in ADHD and normal children. Boxes represent the plus and minus twice the standard error of the mean. The white line represents the mean of the group. Bars above and below the boxes include the entire data set except for outliers whose data are depicted as individual dots.

Generally, results showed a significantly lower scores in the ADHD group in comparison to the control group (*t* (36) = −2.767, *p*=0.009). Although, a significant difference existed between two groups regarding the expressive suppression subset (*t* (1, 36) = −2.37, *p*=0.024), regarding the cognitive reappraisal subset, there was no significant difference between the ADHD and control groups (*t* (1, 36) = −1.27, *p*=0.021).  Figure 4 shows the differences between two groups regarding all three cognitive assessments of RMET, working memory, and emotion regulation.

### 3.5. Correlation Analyses

Results showed that a significant correlation existed between scores of working memory and RMET with BM discrimination in the ADHD group (*r* = 0.442, *p*=0.01, and *r* = 0.403, *p*=0.05, respectively). However, no significant association found between scores of the BM task and emotion dysregulation in the ADHD group (*r* = 0.19, *p*=0.41). Moreover, in the control group, there were significant associations between working memory and BM scores (*r* = 0.522, *p*=0.05). Neither the RMET nor emotion regulation scores were significantly correlated with the BM task in the control group (Table 1).

## 4. Discussion

To the best of our knowledge, it is the first study investigating BM detection by using noisy environments in children with ADHD. Our findings represented that in the presence of visual motion noise, the children with ADHD showed poorer performance in moderate ratios of noise in comparison to TD children. One of the possible mechanisms underlying poorer performances of noisy BM perception in children with ADHD might be the existence of deficits in bottom-up brain processing. In other words, top-down brain processing in the BM task is about extracting and recognizing of that particular human action from several numbers of possibilities while bottom-up brain processing is about gathering visual environmental data about the BM features [14]. Considering the facts that in the current study, BM type was restricted to a single type of motion (walking) and motion noises (which are stimulus-based manipulations added to the stimuli), and we can assume that the need for top-down cognitive processes was minimized in such a design.

Thus, one can hypothesize that the poor performance of children with ADHD in the BM task implicates deficits in the bottom-up brain processing or basic motion perception that support discriminating basic features of BM such as direction.

Interestingly, children with ADHD showed poorer performance in the BM task only in moderate ratio of noise, while in higher noise ratios, the performance of both TD and ADHD groups decreased similarly. It depicts the fact that when motion noise is high, even children with normal perceptual and cognitive development represent shortfalls in direction discrimination, while in moderate ratios of noises, a TD child with intact basic perceptual brain systems can surpass a child with ADHD in direction discrimination tasks. Difficulties in BM detection have also been observed in other psychiatric disorders such as autism and schizophrenia [14, 28]. Interestingly, a bunch of studies found the co-occurrence of autism and ADHD-related symptoms [29–32]. For example, deficits in motor control including neurological soft signs (subtle impairments of sensory integration, motor coordination, and difficulties in sequencing complex motor tasks) are common in both autism and ADHD [33, 34].

As another part of the study, we found the lower scores of emotion regulation, working memory, and RMET in ADHD compared to the TD group. In addition, as a novel finding we showed that performances in the BM task is prominently correlated with performances in working memory and RMET experiments in all participants, particularly the ADHD group. This finding somehow reflects the role of top-down sociocognitive processing in BM perception. There are several investigations representing deficits in working memory and cognitive empathy among individuals with ADHD. For example, Fassbender et al. in an imaging study proposed that children with ADHD demonstrated a reduced working memory task-specific brain activation compared to their peers [35]. Furthermore, there are evidences showing that children with ADHD show low performance in tasks of false belief and a high error rate in attributing mental states and emotions to different features of the face [36, 37]. However, to the best of our knowledge, this is the first study showing association between BM perception and top-down sociocognitive factors in children with ADHD.

According to Baddeley's model, working memory is an essential component for monitoring and modulating entering information by setting the allocation of attention in line with different social and nonsocial behaviors [38]. For example, in a social context, a higher working memory capacity can help a child to take into perspective the situational and social expectancies and therefore modify an emotional reaction [39]. Somehow in line with Baddeley's model, in the current study we represented that working memory capacity is prominently associated with accurate performance in one of the main hallmarks of social cognition called BM processing. Furthermore, alongside accepting, storing, and manipulating information, working memory generates signals that improve the quality of the information that it processes in different social and nonsocial contexts [40, 41]. As an example of mechanisms that working memory administer to improve the quality of information, it is directing the orienting movements of the eyes or other appendages (e.g., hands and other sensory systems) toward an object [42]. Given together, one can hypothesize that working memory as an inextricably inter-related component with attention [39] is necessary for stimuli processing in real-life social scenarios containing different levels of noises. In line with this hypothesis, our findings confirmed that children with ADHD suffering from deficits in working memory capacity might not process noisy social stimuli as high resolution as TD children do with intact working memory.

Moreover, we found association between the RMET and BM score in ADHD but not the TD group. Although there were little data in ADHD, Rice et al. assessed the developmental association between social perception and TOM ability, the two cornerstones of social development in TD children [43]. After controlling for age and IQ, they proposed that perception of noisy BM was significantly correlated with measures of TOM. Moreover, there are different theoretical perspectives showing different points about the link between social perception and TOM ability [43]. For example, one perspective describes that social perception and TOM are domain-specific areas of social development while others propose that these skills may represent a more integrated social system. However, whether social perception (i.e., BM perception), particularly in real noisy environments, and TOM ability reflect two sides of the same coin or are domain-specific areas of social development is still unclear.

Regarding emotion regulation, our findings showed that children with ADHD significantly were poorer at the expressive suppression subset of emotion regulation than TD children. However, we did not find any prominent association between emotion regulation and BM perception in both TD and ADHD groups. This finding may suggest that although emotion dysregulation is one of the main aspects of ADHD, this aspect may not necessarily contribute to difficulties in social perception and interaction in children with ADHD. It may also be due to the fact that our experiment is mostly related to recognition processes rather than emotional data processes.

## 5. Conclusion and Implication of the Findings

In sum, our study showed that children with ADHD have problems with discriminating BM direction in a noisy paradigm. This finding could redraw the therapists, teachers, and parents' attention on the role that different noisy environments such as school classes have on the quality of social interactions in children with ADHD. This may highlight the need for specific designs and requirements for a child with ADHD in order to have more effective social activities in different places. Moreover, our investigation represented that TOM and working memory deficits may underlie difficulties in BM perception in children with ADHD. This may help clinicians to provide novel therapeutic interventions to improve social perception and consequently the low quality and small quantity of the friendship in children with ADHD mentioned by previous studies [6, 44]. In another words, as social impairments are not considered as main difficulties of children with ADHD, clinicians may ignore the importance of therapies on social cognition improvement. This study may have implications in such novel design of interventions encompassing sociocognitive variables such as working memory and TOM. Nevertheless, future studies are required to clarify more the pathological mechanisms of social difficulties in children with ADHD.

## Figures and Tables

**Table 1:** Correlation between biological motion direction discrimination and cognitive tasks.

	BM		WM		RMET		ER	
	*r*	*p*	*r*	*p*	*r*	*p*	*r*	*p*
ADHD								
BM			0.442^*∗*^	0.01	0.403^*∗*^	0.01	0.19	0.41
WM	0.442^*∗*^	0.01			0.595^*∗*^	0.01	0.195	0.143
RMET	0.403^*∗*^	0.01	0.595^*∗*^	0.01			0.232	0.21
ER	0.19	0.41	0.195	0.43	0.232	0.21		

Control								
BM			0.522^*∗*^	0.02	0.332	0.16	−0.26	0.27
WM	0.522^*∗*^	0.02			0.219	0.36	−0.21	0.36
RMET	0.332	0.16	0.219	0.36			0.036	0.88
ER	−0.262	0.27	−0.218	0.36	0.036	0.88		

BM, biological motion; WM, working memory; RMET, Reading the Mind in the Eyes; EM, emotion regulation. ^*∗*^*p* < 0.05.

## Data Availability

All data of this study are available upon request from the first author Samaneh Imanipour.

## Additional Points

One of the main limitations of our study was the lack of BM assessment in a no-noise condition. Although in a pilot assessment, we administered the no-noise condition for each participant to let them know the task environment and nearly all children with ADHD discriminate BM direction correctly, future studies are required to clarify the difference between no-noise and noisy conditions in the BM paradigm in children with ADHD.
